# Epidemiology and Serum Metabolic Characteristics of Acute Myocardial Infarction Patients in Chest Pain Centers

**Published:** 2018-07

**Authors:** Dazhi DENG, Ling LIU, Guangma XU, Jianting GAN, Yin SHEN, Ying SHI, Ruikai ZHU, Yingzhong LIN

**Affiliations:** 1. Dept. of Emergency, The People’s Hospital of Guangxi Zhuang Autonomous Region, Nanning, Guangxi Province, 530021, China; 2. Dept. of Cardiology, The People’s Hospital of Guangxi Zhuang Autonomous Region, Nanning, Guangxi Province, 530021, China

**Keywords:** Epidemiology, Acute myocardial infarction, Metabolomics, Chest pain centers

## Abstract

**Background:**

We aimed to find a potential earlier diagnostic strategy for acute myocardial infarction (AMI) by investigating the epidemiology and serum metabolic characteristics of AMI patients in comparison with those of chest pain controls (CPCS).

**Methods:**

We conducted this prospective, non-randomized, observational study of patients with acute chest pain symptoms presenting to the Emergency Rooms (ER) in The People’s Hospital of Guangxi Zhuang Autonomous Region, Nanning, Guangxi Province, China from January 2015 to July 2016. We included a cohort of 45 patients with AMI together with 45 age- and sex-matched CPCS. The epidemiology of AMI was collected, and the phenotypic characteristics of the serum metabolite composition of AMI patients were determined using a combination of 1H nuclear magnetic resonance (NMR)-based metabolomics and clinical assays.

**Results:**

The epidemiology showed that elderly AMI patients with chest pain syndrome presenting to ER have little awareness of their physical condition and compliance with medication. Significant serum metabolic differences observed between AMI patients and CPCS were highlighted by system differentiations in multiple metabolic pathways including anaerobic glycolysis, gluconeogenesis, tricarboxylic acid cycle (TCA cycle), protein biosynthesis, lipoprotein changes, choline and fatty acid metabolisms and intestinal microbial metabolism.

**Conclusion:**

The epidemiology and serum metabolic phenotypes observed here demonstrated that integration of metabolomics with other techniques could be useful for better understanding the biochemistry of AMI and for potential AMI molecular diagnosis. We should improve the general public’s awareness of AMI, including early symptoms, risk factors, emergency responses, and treatments for related comorbidities.

## Introduction

The chief cause of global mortality remains heart disease related to the aging trend among the world population (1–3). There is an increase of 1.5% in the mortality rate for every 30-minute increase in the reperfusion time for acute myocardial infarction (AMI) (4). Chest pain is the most common symptom of cardiovascular disease (CVD) that causes patients to present in Emergency Rooms (ER) worldwide (5–7). Patients with symptoms suggestive of AMI account for almost 10% of ER consultations globally (8). Therefore, the goal of chest pain centers is focusing on enhanced operational efficiencies in the care of AMI patients (9–11). Patients with continuous or intermittent chest pain have been diagnosed as AMI after coronary arteriography with normal baseline high-sensitivity cardiac troponin immunoassays (Hs-CTnI) values (≤ 99th percentile upper reference limit), which was previously regarded as a biomedical marker of AMI. Others with elevated Hs-CTnI values (> 5×99th percentile upper reference limit) were confirmed as normal coronary artery after coronary arteriography (12, 13). Hs-CTnI assay values in patients with AMI, however, did not change within 3 hours (14), which indicated that it was not an ideal biochemical marker for use by staff in chest pain centers to diagnose non-ST-segment elevated myocardial infarction (NSTEMI) onset within 3 hours. Consequently, there are many controversial medical challenges when making a definitive diagnosis of AMI based on the international consensus (15) when using Hs-CTnI as point of care testing (POCT) for biochemical diagnosis within 30 minutes after arrival to ER (14,16).

A question arises on how to find a better diagnosis strategy for AMI patients? The serum metabolites (or metabolome) can reflect the real-time expression of all biochemical processes in the human body, and abnormal metabolomics changes manifest biochemistry changes in the whole system (17–19).

Although this powerful approach has already been applied to investigate the pathogenesis and diagnosis of various diseases, such as diabetes (20), de novo acute myeloid leukemia (AML) (21) and coronary artery disease (22, 23), to the best of our knowledge, only a limited number of studies have been reported so far for AMI patients in chest pain units. The reported metabolomics investigations of AMI were focused on planned myocardial infarction, which proved the usefulness and feasibility of the NMR-based metabolomics methods as potential noninvasive tools for the prognosis of AMI (24). However, it is very hard to translate the results from a well-controlled study with limited cases to clinical settings in the ER.

The aims of the present study were to evaluate the epidemiology of AMI and define the serum metabolic differences between AMI patients and chest pain controls (CPCS) by identifying potential biomarkers of such metabolic differences for earlier AMI diagnosis. In this study, we reported epidemiologic and serum metabotypic characteristics of a cohort of 45 AMI patients against 45 age- and sex-matched CPCS.

## Materials and Methods

### Cohort Management and Sample Collections

This study was approved by the institutional Review Board of The People’s Hospital of Guangxi Zhuang Autonomous Region, and the use of serum samples together with the epidemiologic records were conducted with informed consent based on the Helsinki declaration.

Serum samples were collected prior to any medications. We conducted this prospective, non-randomized, observational study of patients with acute chest pain symptoms presenting to the ER in this hospital in China from January 2015 to July 2016.

Epidemiologic data of patients, including present, past, family and medication histories, were collected when patients presented in the ER. Within 10 min after first medical contact (FMC), 12-lead electrocardiograms (ECG) were taken with an MECG-300 multiple leads electrocardiograph analysis system (medex-tech, China). Simultaneously, blood samples were collected. In the clinical laboratory, POCT Hs-CTnI, white blood cell count (WBC) and blood C-reactive protein (CRP) concentrations were analyzed using a fluorescence immunity analyzer (Tebsun Bio-tech, China), a 100XN-1000 automatic hematology analyzer (Sysmex, japan) and an i-CHROMA reader fluorescence immunity analyzer (BodiTech Med, Korea), respectively. Blood creatine kinase (CK), creatinine (Cre) and creatine kinase isoenzyme (CK-MB) concentrations were measured with a Cobas Modular P800 automated immunology analyzer (Roche Diagnostics GmbH, Germany). Emergency selective coronary angiography (CAG) was performed with the Allura Xper FD20 interventional X-ray imaging system (Philips, Netherlands) according to the manufacturer’s instructions.

Following FMC, four values were obtained that form our dignostic cornerstones: 12-lead electrocardiogram (ECG) in 10 minutes, POCT Hs-CTnI in 20 minutes, thrombolysis in myocardial infarction (TIMI) risk score in 30 minutes (Table 1) (25) and Grace risk score in 60 minutes (Table 2) (26).

**Table 1:** TIMI risk score for patients with chest pain symptoms in the ED

***Characteristics***	***Score***	***AMI***	***CPCS***	***P-value***	***Details***
Age ≥65 yr	1	1(0–1)	0(0–1)	0.142	
At least 3 risk factors for CAD	1	0(0–1)	0(0–0)	0.001	family history of CAD hypertension hypercholesterolemia diabetes mellitus being a current smoker
Use of aspirin in last 7 days	1	0(0–0)	0(0–0)	1	
Severe anginal symptoms	1	1(0–1)	0(0–0)	0.000	≥2 anginal events in last 24 h
Elevated serum cardiac markers	1	1(0–1)	0(0–1)	0.294	CTnI of POCT
ST deviation	1	1(0–1)	0(0–0)	0.000	≥0.5 mm
Significant coronary stenosis	1	0(0–0)	0(0–0)	1	prior coronary stenosis ≥50%
Total score	0–2 low risk, 3–4 middle risk, 5–7 high risk				

Continuous variables were shown as mean ± SD; skewed distribution variables were shown as median (minimum, maximum)

**Table 2:** Grace risk score for patients with chest pain symptoms in the ED

	***Age (points)***	***HR (points)***	***SBP (points)***	***Cr (points)***	***Killip class (points)***	***Dichotomous factors (points)***
	≤30 (0)	≤50 (0)	≤80 (58)	≤34(1)	I (0)	Cardiac arrest at admission (39)
	30–39 (8)	50–69 (3)	80–99 (53)	35–69(4)	II (20)	ST-segment deviation (28)
	40–49 (25)	70–89 (9)	100–119 (43)	70–105 (7)	III (39)	Elevated cardiac enzymes (14)
	50–59 (41)	90–109 (15)	120–139 (34)	106–140(10)	IV(59)	
	60–69 (58)	110–149(24)	140–159(24)	141–175(13)		
	70–79(75)	150–199(38)	160–199(10)	175–352(21)		
	≥80 (91)	≥200 (46)	≥200 (0)	>353(28)		
AMI	58 (8–91)	9(0–15)	24(0–53)	7(4–28)	0(0–59)	28(0–42)
CPCS	58(25–91)	9(3–24)	34(10–53)	7(4–10)	0(0–0)	0(0–14)
*P*-value	0.908	0.060	0.330	0.345	0.000	0.000

Skewed distribution variables were shown as median (minimum, maximum). Total score = age + HR + SBP + Cr + Killip class + cardiac, arrest + ST-segment and deviation + elevated cardiac enzymes. Low-risk scores range from 1 to 88, intermediate-risk scores range from 89 to 118, and high-risk scores are ≥119. HR: heart rate; SBP: systolic blood pressure; Cre: creatinine in μmol/L

Therefore, the criteria for AMI evaluation included 1) symptoms of continuous or intermittent chest pain; 2) with or without ischemic changes in ECG; 3) with or without elevated CTNI of POCT; 4) with elevated myocardial enzymes of CK and CK-MB; 5) coronary infarction or coronary stenosis ≥ 50% shown in subsequent coronary angiography after FMC. CPCS inclusion criteria included 1) symptoms of chest pain; 2) without ischemic changes in ECG; 3) without elevated myocardial enzymes of CK and CK-MB; and 4) no return visits because of chest pain symptoms within 4 months after being released from the ED. The exclusion criteria include 1) those unable or unwilling to consent; and 2) those who had been diagnosed with a malignant tumor, autoimmune disorders, severe infectious diseases, aortic dissection, pulmonary embolism, trauma, myocarditis, liver dysfunction, etc.

In the current study, the selected cohort was composed of 45 AMI patients and 45 age- and sex-matched CPCS. Serum samples for later metabolomics analysis were collected in a standard procedure and stored in a −80 °C freezer.

### 1H NMR Spectra of Serum Samples

Each serum sample (200 μL) was mixed with 400 μL saline solution (0.9% NaCl, w/v) containing 50% D2O (as a field lock). After vortex and centrifugation for 10 min (11180 × g, 4 °C), 550 μL of the supernatant of each sample was transferred into a 5 mm nuclear magnetic resonance (NMR) tube. NMR analysis of serum samples were performed at the NMR laboratory of Wuhan Institute of Physics and Mathematics, the Chinese Academy of Sciences, in the form of paid service.

All 1H NMR spectra were recorded at 298 K on a Bruker AVIII 600 spectrometer (operating at 600.08 MHz for 1H and at 150.93 MHz for 13C) equipped with a cryogenic inverse detection probe (Bruker Biospin, Germany). To attenuate the signals from macromolecules such as lipoprotein in serum, a 1H NMR spectrum was acquired for each sample using the standard Carr-Purcell-Meiboom-Gill (CPMG) pulse sequence (RD-90°-(τ-180°-τ) n-acquisition), as previously described (27). The 90° pulse length was set to approximately 10 μs for each sample and the water signal was suppressed with weak continuous wave irradiation during recycle delay (RD). Data points (32 K) were collected for each spectrum with a spectral width of 20 ppm and RD of 2 s. The spin-spin relaxation delay, 2nτ, was set to 100 ms. Free induction decays (FIDs) for all samples were multiplied by an exponential function with a line broadening factor of 0.3 Hz prior to Fourier transformation. Chemical shifts for all spectra were then manually referenced to the anomeric proton signal of α-glucose (δ 5.233).

For the purposes of signal assignments, a series of two-dimensional NMR (2D NMR) spectra were recorded and processed for selected samples, as previously described (28). These spectra included 1H–1H correlation spectroscopy (COSY), total correlation spectroscopy (TOCSY), 1H–13C heteronuclear single quantum correlation (HSQC) and 1H–13C heteronuclear multiple bond correlation (HMBC) spectra.

### NMR Data Processing and Multivariate Data Analysis

All 1H NMR spectra were manually corrected for phase and baseline distortions using Topspin (V3.0, Bruker Biospin), and the spectral regions at δ 0.5–9.5 were divided into buckets with equal width of 0.004 ppm (2.4 Hz) using the AMIX software package (V3.8.3, Bruker Biospin). The regions at δ 4.60–5.15 and δ 5.5–6.0 were discarded to eliminate the effects of imperfect water saturation and to remove urea signals.

Multivariate data analysis was conducted with SIMCA-P+ package (V12.0, Umetrics, Sweden) following normalization to the volume of serum samples. Principal component analysis (PCA) was carried out on the mean-centered data to generate an overview and check for the outliers. Partial-least-squares discriminant analysis (PLS-DA) and the orthogonal projection to latent structure with discriminant analysis (OPLS-DA) were subsequently performed using the unit-variance scaled data to find metabolites having significant intergroup differences (29). The OPLS-DA models were built with two components calculated with 6-fold cross-validation. These models were further evaluated for their validities using the CV-ANOVA method (30). After back-transformation, the loadings were plotted using an in-house developed MATLAB (V7.1, The Mathworks, MA) script with correlation coefficients color-coded for each variable (or the metabolite signals). The color-coded variables indicate the significance of metabolites contributing to the intergroup differentiation, with a “hot” colored (e.g., red) metabolite being more significant than a “cold” colored (e.g., blue) one. Cutoff values for the correlation coefficients were chosen depending on the number of samples used to extract metabolites having significant intergroup differences based on the discrimination significance (*P* < 0.05) for the Pearson’s product-moment correlation coefficients (21). In this study, a cutoff value of |r| > 0.288 (r > +0.288 and r < −0.288) was chosen for the correlation coefficient as significant based on the discrimination significance (*P* < 0.05).

## Results

### Epidemiology of AMI Patients

The AMI group exhibited skewed distribution variables of TIMI risk score, which were shown as the median (minimum, maximum), including 1 (0–1) for age, 0 (0–1) for risk factor, 0 (0–0) for use of aspirin, 1 (0–1) for severe angina symptoms, 1 (0–1) for elevated serum cardiac markers, 1 (0–1) for ST deviation and 0 (0–0) for significant coronary stenosis. However, the chest pain control group exhibited 0 (0–1) for age, 0 (0–0) for risk factor, 0 (0–0) for use of aspirin, 0 (0–0) for severe angina symptoms, 0 (0–1) for elevated serum cardiac markers, 0 (0–0) for ST deviation and 0 (0–0) for significant coronary stenosis (Table 1). Details of skewed distribution variables of Grace Risk score were shown in Table 2. The AMI group exhibited 58 (8–91) for age, 9 (0–15) for heart rate, 24 (0–53) for systolic pressure, 7 (4–28) for creatinine, 0 (0–59) for Killip class (points), and 28 (0–42) for dichotomous factors. The chest pain control group exhibited 58 (25–91) for age, 9 (3–24) for heart rate, 34 (10–53) for systolic pressure, 7 (4–10) for creatinine, 0 (0–0) for Killip class, and 0 (0–14) for dichotomous factors, respectively.

**Fig. 1:**
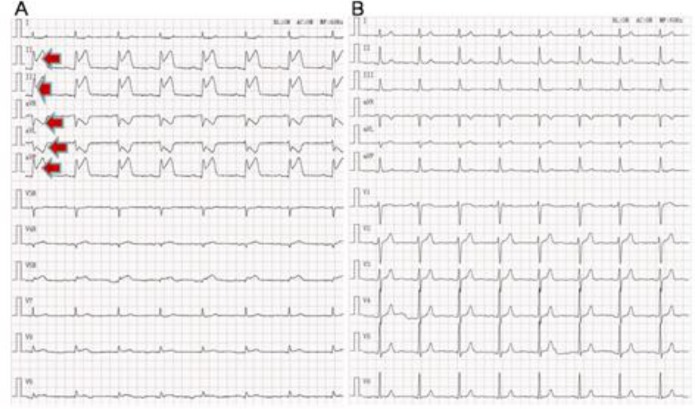
Typical ECG of AMI (A) and chest pain control (B), arrows showed significant ST-Segment-T wave changes ≥0.5 mm in contiguous leads, including anterior leads (II, III AVF), lateral/apical leads (I, AVL), which indicating infarction in right coronary artery

**Fig. 2:**
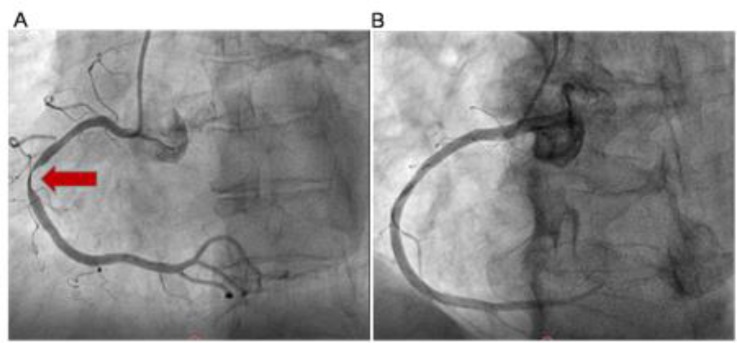
Typical CAG result of AMI (A) and CPCS (B). Arrows denoted cardiac coronary stenosis ≥95%, which indicating thrombus in the right coronary artery

Compared with the chest pain control group, significantly higher TIMI scores (3 versus 1) and Grace risk score (131 versus 102) in the AMI group were obtained and located, respectively, in middle risk and high risk for smaller scores of aspirin-use history and risk factors for CAD. However, no statistical significance was observed in POCT CTnI, high blood pressure, accelerated heart rate and broad-range chest pain onset time between AMI group and CPCS (Table 3). The median of POCT CTnI (0.13 ng/L) in the AMI group was obviously higher than in the controls (0.09 ng/L), and the median of chest pain onset time in the AMI group (4 h) was remarkably shorter than that in controls (12 h) (Table 3). The chest pain onset time in the AMI group ranged from 20 minutes to 7 days, and one extraordinary AMI patient was undergoing an annual ECG examination in Center of Health Examination (Table 3). Most of the AMI patients exhibited significant changes in the ST-segment-T wave (≥ 0.5 mm) in the ECG (Fig. 1), remarkable elevation values of cardiac biomarkers, including Hs-CTnI of POCT, CK-MB and CK, an obvious rise of inflammatory factors (WBC and CRP) and typical intracoronary thrombus in angiography (Fig. 2). Most of these patients were older males (86.7% vs. 71.1%) with comorbidity such as hypertension (53.33% vs. 48.89%), diabetes mellitus (28.89% vs. 26.67%), hypercholesterolemia (17.78% vs. 20%) and prior ischemic stroke (15.56% vs. 17.78%). A few of them, however, were aware of their physical condition and had taken medication (<7%) as advised by a medical doctor previously.

**Table 3:** Demographics of study cohorts

***Characteristics***	***AMI (n=45)***	***Control (n=45)***	**P*-Value***
Age (yr)	62.600±12.617	61.889±12.454	0.789
Male (%)	86.7	71.1	0.071
Chest pain onset time (h)	4.000(0.30–168.00)	12.000(0.50–240.00)	0.036
TIMI risk score	3.000(1.00–5.00)	1.000(0.00–2.00)	0.000
Grace risk score	131(75–211)	102(59–167)	0.000
**Blood pressure (mm Hg)**			
Systolic	144.000(96.00–204.00)	138.000(95.00–181.00)	0.305
Diastolic	84.756±17.186	77.956±15.920	0.055
Heart rate (beats/min)	76.911±17.777	84.044±13.789	0.036
**Comorbidity**			
Diabetes mellitus (%)	28.89	26.66	0.8139
Chronic kidney disease (%)	8.89	11.11	0.7253
Hypertension (%)	53.33	48.89	0.8330
Atrial fibrillation (%)	4.44	6.67	0.6454
Prior ischemic stroke (%)	15.56	17.78	0.7773
Hypercholesterolemia (%)	17.78	20	0.7877
**Medication**			
ACEI or ARB (%)	2.22	0	/
Beta-blocker (%)	2.22	0	/
Calcium antagonist (%)	2.22	0	/
OHA/insulin (%)	6.67	0	/
**Laboratory data**			
Hs-CTnI of POCT (ng/ml)	0.130(0.03–30.00)	0.090(0.05–0.50)	0.084
WBC (10E9/L)	10.032±3.503	6.988±1.689	0.000
CRP (mg/L)	12.205(0.50–197.44)	0.9000(0.50–8.59)	0.000
Cr (μmoI/L)	91.000(57.00–757.00)	82.000(46.00–131.00)	0.150
CK (ng/ml)	234.100(21.00–3000.00)	33.400(21.00–256.40)	0.000
CK-MB (ng/ml)	37.090(1.42–300.00)	1.410(0.43–6.06)	0.000

Continuous variables were shown as mean ± SD; categorical variables were shown as percentages, skewed distribution variables were shown as median (minimum, maximum). ACEI: angiotensin-converting enzyme inhibitor; OHA: oral hypoglycemic agents; Cr: creatinine; CK: creatine kinase; CK-MB: creatine kinase isoenzyme

### ^1^H NMR Spectroscopy of Serum Samples

To focus on the analysis of small metabolites in human serum, the T2-edited NMR spectra from the CPMG sequence was employed for metabolomics analysis.

**Fig. 3:**
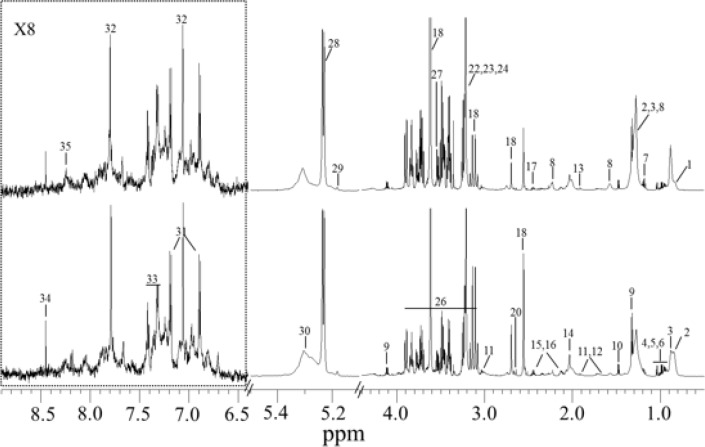
Typical ^1^H CPMG NMR spectra of serum from CPCS and acute myocardial infarction patients. Metabolite keys: 1. High-density lipoprotein (HDL); 2. Low-density lipoprotein (LDL); 3. Very low-density lipoprotein (VLDL); 4. Isoleucine; 5. Leucine; 6. Valine; 7. D-3-hydroxybutyrate (3-HB); 8. Lipid; 9. Lactate; 10. Alanine; 11. Lysine; 12. Arginine; 13. Acetate; 14. N-acetyl-glycoproteins; 15. Glutamate; 16. Glutamine; 17. Acetylcarnitine; 18. EDTA; 20. Citrate; 22. Choline; 23. Phosphocholine (PC); 24. Glycerophosphocholine; 26. Glucose/amino acids; 27. myo-inositol; 28. α-glucose; 29. Triglyceride; 30. Unsaturated fatty acids; 31. Tyrosine; 32. Histidine; 33. Phenylalanine; 34. Formate; 35. Hypoxanthine

The serum ^1^H NMR spectra (Fig. 3) showed that a set of metabolite signals was observable for CPCS and AMI patients. The NMR signals were assigned to individual metabolites based on the published data (27, 30, 31) and were further confirmed individually based on the 2D NMR data (Table 4). Visual inspection showed clear differences in lipid and glucose between the spectra of the control and AMI patient (Fig. 3).

**Table 4:** NMR data and assignments for the metabolites in human serum

***Key***	***Metabolites***	***Moieties***	***δ ^1^H (ppm) and multiplicity^a^***	***δ ^13^C (ppm)***
1	HDL	CH_3_	0.82(m)	#
2	LDL	CH_3_	0.85(m)	#
3	VLDL	CH_3_	0.88(m)	#
4	Isoleucine	αCH, βCH, γCH_3_, δCH_3_	3.65(d), 1.95(m), 0.99(t), 1.02(d)	62.6, 38.8, 17.8, 13.9
5	Leucine	αCH, βCH, γCH_3_, δCH_3_	0.94(d), 3.72(t), 1.96(m), 0.91(d)	24.5, 42.8, 27.3, 24.5
6	Valine	αCH, βCH, γCH_3_	2.26(m), 0.98(d), 1.04(d)	63.4, 31.9, 19.5, 20.9
7	D-3-hydroxybutyrate	CH, CH_2_, γCH_3_, CH_2_	4.16(dt), 2.41(dd), 1.20(d), 2.31(dd)	68.8, 49.5, 24.4, 49.5
8	Lipid	CH_3_, (CH_2_)_n_, CH_2_-C=C, CH_2_-C=O,C-CH_2_-C=, CH=CH-	0.89(m), 1.27(m), 2.0(m), 2.3(m), 2.78(m), 5.3(m)	#
9	Lactate	αCH, βCH_3_	4.11(q), 1.32(d)	63.4, 71.1
10	Alanine	αCH, βCH_3_	3.77(q), 1.48(d)	53.9/178.9, 19.3
11	Lysine	αCH, βCH_2_, γCH_2_, δCH_2_	3.76(t), 1.89(m), 1.72(m), 3.01(t)	57.4, 33.0, 29.4, 42.4
12	Arginine	CH_2_, CH_2_, CH_2_, CH	1.68(m), 1.90(m), 3.23(t), 3.76(t)	26.6, 30.3, 57.2, 160.1
13	Acetate	CH_3_	1.91(s)	26.5/184.4
14	N-acetyl-glycoproteins	CH_3_	2.03(m)	#
15	Glutamate	αCH, βCH_2_, γCH_2_	2.06(m), 2.11(m), 2.36(m)	28.9, 33.4, 57.1
16	Glutamine	αCH, βCH_2_, γCH_2_	2.15(m), 2.44(m), 3.77(m)	30.1, 30.1, 36.4
17	Acetylcarnitine	CH_3_C=O, αCH, αCH′	2.46(m), 2.63(m), 3.90 (m)	#
18	EDTA	CH, CH, CH	2.55(s), 2.68(s), 3.11(q), 3.61(s)	#
20	Citrate	CH_2_(1/2), CH_2_(1/2)	2.52(d), 2.64(d)	48.5, 78.2, 181.7
22	Choline	N(CH_3_)_3_, OCH_2_, NCH_2_	3.2(s), 4.05(t), 3.51(t)	56.5, 58.1, 7.01
23	Phosphocholine (PC)	N(CH_3_)_3_, OCH_2_, NCH_2_	3.22(s), 4.21(t), 3.61(t)	57.1, 74.9
24	Glycerophosphocholine	N(CH_3_)_3_, OCH_2_, NCH_2_	3.22(s), 4.32(t), 3.68(t)	57.1, 74.9
26	Glucose/amino acids	α-CH resonances	3.2–3.9	#
27	*myo*-inositol	1,3-CH, 2-CH, 4,6-CH	3.65(m), 3.29(m), 3.57(m)	#
28	α-glucose	1-CH	5.23(d)	94.8
29	Triglyceride	CH	5.16	#
30	Unsaturated fatty acids	CH, CH	2.73(m), 6.53(m)	137.6
31	Tyrosine	CH, CH	6.89(dd), 7.18(dd)	119.1, 133.3
32	Histidine	2-CH, 4-CH	7.75(t), 7.08(d)	118.1, 136.1
33	Phenylalanine	Ring-CH	7.40(m), 7.33(m), 7.35(m)	132.2, 132.3, 131.1
34	Formate	CH	8.45(s)	151.8
35	Hypoxanthine	CH, CH	8.19(s), 8.21(s)	148.3, 144.6

a Key: s, singlet; d, doublet; t, triplet; q, quartet; m, multiplet; dd, doublet of doublet.

# Undetermined

### Metabotypic characteristics of AMI Patients

Multivariate data analysis of the NMR spectra was performed to reveal the different metabolic patterns between CPCS and AMI patients.

**Fig. 4:**
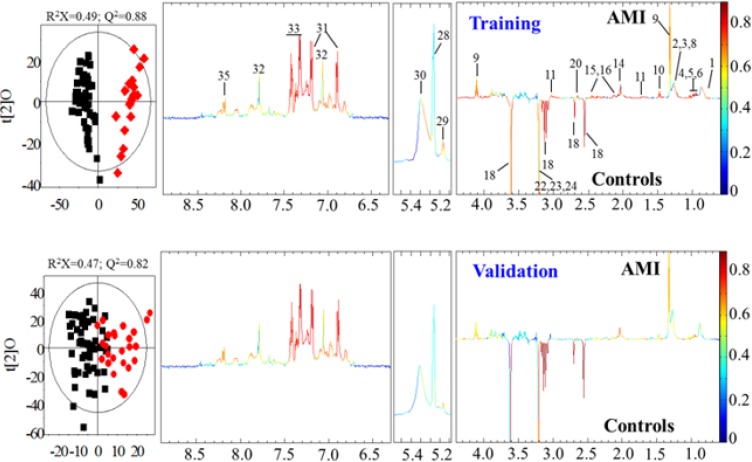
OPLS-DA scores (left) and loadings plots for (A) the training set with CPCS (n = 45, black) and AMI patients (AMI, n = 22, red) (p = 2.1 × 10^−6^ from CV-ANOVA); (B) the validation set with CPCS (n = 45, black) and AMI patients (AMI, n = 23, red) (p = 1.944× 10^−6^ from CV-ANOVA). The results were from the 6-fold cross-validated models, and colored scales were for the correlation coefficients (|r|) of variables

In OPLS-DA models, both age and sex-matched chest pain controls and AMI patients were respectively divided into two subgroups, namely, the training and validation sets (Table 5), to further ensure the model qualities. Two separate models were calculated with the data from the training sets (Fig. 4A) and validation sets (Fig. 4B) so that the latter was also considered as an independent validation to the former. The results from CV-ANOVA showed good qualities for these OPLS-DA models. The validities of these OPLS-DA models indicated that significant differences were present in the serum metabolomic phenotypes between CPCS and AMI patients. Corresponding loadings plots (Fig. 2) revealed significant differences in the levels of several serum metabolites between these two groups (Table 5). Compared with the controls, AMI patients exhibited higher levels in HDL, LDL, VLDL, isoleucine, leucine, valine, lipid, alanine, lactate, lysine, N-acetyl-glycoproteins, glutamate, glutamine, citrate, α-glucose, triglyceride, unsaturated fatty acids (UFA), tyrosine, histidine, phenylalanine and hypoxanthine but lower levels in choline, phosphorylcholine (PC) and glycerophosphorylcholine (GPC).

**Table 5:** Significant differences of serum metabolites between AMI patients and CPCS

***Keys***	***Metabolites***	***Changes in AMI patients against CPCS***	***Correlation coefficients^a^ (R^2^X = 0.49, Q^2^ = 0.88)***
1	HDL	↑	0.74
2	LDL	↑	0.38
3	VLDL	↑	0.38
4	Isoleucine	↑	0.78
5	Leucine	↑	0.78
6	Valine	↑	0.76
8	Lipid	↑	0.72
9	lactate	↑	0.69
10	Alanine	↑	0.79
11	Lysine	↑	0.81
14	N-acetyl-glycoproteins	↑	0.82
15	Glutamate	↑	0.77
16	Glutamine	↑	0.77
20	Citrate	↑	0.68
22	Choline	↓	−0.66
23	Phosphocholine (PC)	↓	−0.66
24	Glycerophosphocholine	↓	−0.66
28	α-glucose	↑	0.39
29	Triglyceride	↑	0.64
30	UFA	↑	0.60
31	Tyrosine	↑	0.79
32	Histidine	↑	0.63
33	Phenylalanine	↑	0.81
35	Hypoxanthine	↑	0.67

a Correlation coefficients, positive and negative signs indicated positive and negative correlation in the concentrations, respectively. The values p= 0.05, |r|=0.38 were used as the corresponding cutoff values of the correlation coefficient for statistical significance based on the discrimination significance, respectively. “↑” and “↓” means the increased and decreased metabolites in AMI patients against CPCS

## Discussion

AMI patients, especially elderly individuals, had little awareness of their physical condition and poor compliance with medications; these patients had middle risk scores in the TIMI score system and a broad range of chest pain onset time. Chest pain symptom was acute stress, which activating the protection mechanism of the hypothalamus-pituitary-adrenal cortex axis and the sympathetic nervous system with high blood pressure and accelerated heart rate. Therefore, almost all elderly individuals in the ER exhibited high risk scores in the Grace Risk score system. Elderly AMI patients presenting to the ER with undifferentiated chest pain always had many comorbidities, such as hypercholesterolemia, hypertension, diabetes, and chronic kidney disease, which are all AMI risk factors that eventually damage the coronary arteries. Fortunately, most of the AMI patients had significant ischemic changes in ECG and markedly elevated myocardial enzymes. We still can use the TIMI risk score system to stratify risk rapidly in patients presenting to ER and use the Grace Risk score system to stratify eventual risk. One of most important missions of the chest pain centers in China is to improve the general public’s awareness of early symptoms, risk factors of AMI and emergency responses to AMI as in other countries (32).The remarkable differences of median of chest pain onset time (AMI 4 h vs. controls 10 h) and the obvious rise of inflammatory factors (WBC and CRP) for AMI patients indicated that chest pain was severe acute stress that affected the immune system mainly by inducing changes in the organism through catabolism. To obtain metabolic details for AMI patients, here we presented serum metabolic pattern in the context of AMI at the ER. The results revealed that AMI patients exhibiting very different serum metabolic signatures from the age- and sex-matched CPCS.

The metabolic differences were highlighted in multiple metabolic pathways involving metabolisms of fatty acids, choline, phenylalanine, intestinal microbial flora, protein biosynthesis and energy. Differential metabolite levels in the sera of AMI patients compared with the chest pain controls clearly indicated a shift of energy metabolism under the condition of AMI. Significantly increased levels of lipoproteins such as HDL, LDL, and VLDL, and lipids such as triglycerides and unsaturated fatty acids in the blood of AMI patients indicated that a number of lipids accumulated in the blood vessel are probably increasing the risk of atherosclerosis. These results concerning lipid metabolism reported here were also in agreement with aforementioned AMI risk factors such as hypertension and coronary heart disease (33–35).

Lower levels of membrane moieties, such as choline, phosphatidylcholine (PC) and GPC, in the serum of AMI patients suggested disruption of cell structural integrity since they are essential elements for structural integrity of cell membranes. Importantly, phosphatidylcholine is metabolized by intestinal microbiota to produce the proatherogenic species, choline and trimethylamine oxide (TMAO), which was found to be associated with gut microbiota metabolism (36). Previous studies showed that the significant depletion of PC and choline was related to the development of CAD (37, 38) and aberrant intestinal microbial metabolism (39). Supportive evidence of disruption of microbiota could also be found in the significantly elevated levels of phenylalanine and tyrosine in the serum of AMI. Phenylalanine is an essential amino acid for all mammals, and its dietary intake is essential for protein biosynthesis. It can then be metabolized into tyrosine and tryptophan by hydroxylation. Previous studies showed that the perturbed phenylalanine metabolism was associated with a microbial fermentation process, in which dietary fiber contains choline and phenylalanine (40). Collectively, these metabolomics results indicated that AMI was deterioration of CAD and highly associated with intestinal microbiota metabolism. Differential metabolite levels in the sera of AMI patients compared with those of the chest pain controls clearly indicated a shift of energy metabolism under the condition of stress. In the current study, higher levels of serum glucose and lactate in AMI patients than those in the controls suggested that accelerated gluconeogenesis and altered anaerobic glycolysis processes occurred. Previous studies reported that a higher serum glucose level may be associated with insulin-resistance and the development of metabolic syndrome including obesity and type 2 diabetes (41). Of note, the level of hypoxanthine, the sequential purine degradation product involved in anaerobic glycolysis process, was also higher in the serum of AMI patients than in the chest pain controls, further confirming the observations that altered anaerobic glycolysis and energy metabolism. Serum hypoxanthine under the stress condition of AMI might be regarded as a biomarker.

Interestingly, higher levels of citrate and amino acids, including alanine, glutamine, histidine, valine, and isoleucine, in the serum of AMI patients indicated that the TCA cycle was enhanced and probably fed by those amino acids. In addition, such elevated levels of amino acids also suggested degradation of lipoproteins and disruption of energy metabolism in AMI patients. However, it remains unknown whether and how these confounding factors contribute to the aforementioned metabolic differences, although age- and sex-matched controls were used to eliminate the effects of some confounding factors in this study. Further studies that exclusively factor in specific comorbidities, such as hypercholesterolemia, hypertension and diabetes, are needed to further refine our preliminary findings.

## Conclusion

This study has proved the feasibility for the NMR-based metabolomics approach to distinguish the serum metabolic profiles of AMI patients from those of chest pain controls manifested by aberrant metabolism pathways, including glycolysis/gluconeogenesis, TCA cycle, choline and fatty acid metabolisms and intestinal microbial metabolism. These findings provided a better understanding the epidemiology and potential molecular diagnosis of AMI. We should improve the general public’s awareness of early symptoms, risk factors of AMI, emergency responses to AMI and the treatment of comorbidities of AMI.

## Ethical considerations

Ethical issues (Including plagiarism, informed consent, misconduct, data fabrication and/or falsification, double publication and/or submission, redundancy, etc.) have been completely observed by the authors.
